# On the generalizability of resting-state fMRI machine learning classifiers

**DOI:** 10.3389/fnhum.2014.00502

**Published:** 2014-07-29

**Authors:** Wolfgang Huf, Klaudius Kalcher, Roland N. Boubela, Georg Rath, Andreas Vecsei, Peter Filzmoser, Ewald Moser

**Affiliations:** ^1^Center for Medical Physics and Biomedical Engineering, Medical University of ViennaVienna, Austria; ^2^MR Centre of Excellence, Medical University of ViennaVienna, Austria; ^3^Department of Statistics and Probability Theory, Vienna University of TechnologyVienna, Austria; ^4^Department of Radiodiagnostics and Nuclear Medicine, Medical University of ViennaVienna, Austria; ^5^Department of Pediatrics and Adolescent Medicine, St. Anna Children's Hospital, Medical University of ViennaVienna, Austria; ^6^Department of Psychiatry, University of Pennsylvania Medical CenterPhiladelphia, PA, USA

**Keywords:** machine learning, classification, resting-state fMRI, regional homogeneity, support vector machines

## Abstract

Machine learning classifiers have become increasingly popular tools to generate single-subject inferences from fMRI data. With this transition from the traditional group level difference investigations to single-subject inference, the application of machine learning methods can be seen as a considerable step forward. Existing studies, however, have given scarce or no information on the generalizability to other subject samples, limiting the use of such published classifiers in other research projects. We conducted a simulation study using publicly available resting-state fMRI data from the 1000 Functional Connectomes and COBRE projects to examine the generalizability of classifiers based on regional homogeneity of resting-state time series. While classification accuracies of up to 0.8 (using sex as the target variable) could be achieved on test datasets drawn from the same study as the training dataset, the generalizability of classifiers to different study samples proved to be limited albeit above chance. This shows that on the one hand a certain amount of generalizability can robustly be expected, but on the other hand this generalizability should not be overestimated. Indeed, this study substantiates the need to include data from several sites in a study investigating machine learning classifiers with the aim of generalizability.

## 1. Introduction

One of the main goals in analyzing fMRI data lies in the exploration of potential clinical application, like the use of fMRI data for diagnostic purposes (Fu and Costafreda, 2013; Wager et al., 2013; Welsh et al., 2013). To this end, differences in brain functioning are explored at single-subject level, with the aim of finding a reliable classifier that can differentiate between two or more subject groups (e.g., patients with a particular disorder vs. healthy controls or patients with different disorders). Employing and training such classifiers is one of the fields of machine learning, others being regression, unsupervised learning (Zeng et al., 2014), etc. The use of classifiers on fMRI data, however, is still in its infancy; while promising first results on clinical samples exist (Koutsouleris et al., 2009; Shen et al., 2010; Arbabshirani et al., 2013; Zeng et al., 2014), classifiers cannot yet be deployed in practice. Due to the inherent complexity of the problems under consideration (e.g., biological vs. clinical homogeneity of subject groups), current experiments are more often focused on non-clinical research questions as proofs-of-concept. Among the toy research questions more often tackled are classification of sex (Wang et al., 2012), age (Dosenbach et al., 2010; Vergun et al., 2013), or other clearly identifiable target variables (Tagliazucchi et al., 2012; Ash et al., 2013).

In contrast to other widely employed approaches to fMRI data analysis, which focus on group characteristics and group statistics, the training of a classifier relates to the analysis of individual datasets, and its results allow quantification (of e.g., diagnostic group membership) on a single-subject level (Pereira et al., 2009; Lemm et al., 2011; Lee et al., 2013). Such quantifications are inherently more difficult to be achieved due to lower power compared to group analysis which means that even where significant group differences can be found, classification of single-subject datasets may not always be possible [and indeed rarely is with the commonly employed study sample size of about 20 subjects (Murphy and Garavan, 2004) per group].

Another challenge of training a classifier for clinical use rests in the heterogeneity of measurement protocols: a classifier that works with a particular fMRI task measured on a particular scanner hardware (and under other particular circumstances) may not work as well if one or more of these parameters are changed. In particular, a classifier can only be of use for an application in clinical practice if it is not too dependent on the task performance of each subject, which might prove difficult to control for. Overall, this means that the scan protocol should be as standardized as possible and the outcome measures as insensitive to subject task performance or hardware specifics as possible.

A promising approach to standardizing the scan protocol may be task-free resting-state fMRI (RS-fMRI). In this fMRI paradigm, subjects are expected to lie still in the scanner without doing or thinking anything in particular, making it universally applicable. Despite the apparent lack of constraints, functional patterns observed during rest are remarkably robust (Fox and Raichle, 2007; Biswal et al., 2010; Kalcher et al., 2012) and data acquired at rest might thus be amenable to a classifier intended to be generalizable to different studies or different research groups or both. Still, because of the complex and not yet fully understood nature of resting-state fluctuations (Lecrux and Hamel, 2011; Boubela et al., 2014; Kalcher et al., 2014), multiple options for using these data as features for machine learning classifiers exist (Shen et al., 2010; Haxby, 2012; Vergun et al., 2013). A general comparison of different features has not yet been performed but there exist some results on particular resting-state based classification features.

A recent result of a machine learning classifier on resting-state fMRI data was presented by Wang et al. (2012) who used a measure termed regional homogeneity (Zang et al., 2004) based on local synchronicity of BOLD timecourses, and achieved a classification accuracy between men and women of about 83%. For our purposes, one relevant aspect of this publication is that both the methodology employed and the dataset used are publicly available, thus allowing for the use of the results published as a baseline for a comparison of wider generalizablility of similar machine learning classifiers. Another aspect is that the classifier features used are easily replicable and naturally interpretable as the homogeneity of the blood flow in various areas of the brain. Finally, the intra-study classification accuracy on the test set, which is around 80%, together with the use of a linear classifier in the (only linearly transformed) original data space, making it plausible that the risk of the result reflecting overfitting to the study dataset is low and generalizability of results is thus probably higher.

A couple of questions concerning the generalizability of machine learning classifiers have already been approached, but there are relatively few methodological publications on machine learning in fMRI examining this aspect (Demirci et al., 2008). The first test of the generalizability of a classifier can be found in its capability to classify subjects from the same dataset as those used for the training of the classifier, but which were not themselves part of the training dataset. This represents a common class of methods (termed resampling methods) for evaluating model stability, most notably cross validation (Hastie et al., 2009). While this is a valid method for evaluating classifier accuracy on an independent dataset (independent as far as different observations are used) there might still be some amount of bias due to non-independence between datasets of one study due to common factors such as characteristics of the study population and the specifics of the hardware used for scanning.

To this end, the accuracy of a classifier trained on one dataset must be tested on an independent dataset originating from one or more different though similar studies. Here, we will use two types of test datsets fulfilling these requirements, the first being single-study datasets separate from the training study datasets, and the second being datasets pooled from multiple different studies. The single-study datasets can be used to estimate the accuracy one can expect when applying published classifiers to one's own study's data, the results on pooled datasets can be viewed to represent the accuracy that can be achieved with a particular classifier used on a wider population (e.g., with regard to broader clinical use).

Similar considerations apply to the selection of training datasets. First, classifiers trained on single-study datasets are used to yield an estimate of how generalizable single-study results might be on wider populations. Second, classifiers trained on data pooled from multiple centers are investigated to evaluate whether these can achieve higher generalizability than classifiers trained on single-study datasets.

In this study, we aimed to examine the generalizability of machine learning classifiers trained on single-study as well as on pooled multi-center fMRI datasets. The results presented here (1) provide a baseline for generalizability of machine learning classifiers presently in use and (2) offer directions for the investigation of future classifiers and features yielding increased classification accuracy on a general population.

## 2. Methods

Data for simulations were downloaded from the 1000 Functional Connectomes (FCon) and COBRE projects, and all samples of healthy adult subjects with available demographics, anatomical and functional data (otherwise classification could not be performed) were included in the analysis. This means that the datasets AnnArbor_a, Milwaukee_a, NewYork_a, NewYork_a_ADHD, Ontario, Taipei_a, and Taipei_b as well as the patient group from the COBRE dataset were excluded. In addition, six subjects from the Beijing_Zang dataset were excluded because the readme file of the study indicated potential technical problems with those subjects' data, leaving 1170 single-subject datasets for analysis (for details, see Table 1).

**Table 1:** **Overview on studies and number of subjects included in the analysis**.

	**Total**	**Female**	**Male**	**Age**	***SD***
AnnArbor_b	35	19	16	47.6	26.3
Atlanta	28	15	13	30.9	9.9
Baltimore	23	15	8	29.3	5.5
Bangor	20	0	20	23.4	5.3
Beijing_Zang	192	118	74	21.2	1.8
Berlin_Margulies	26	13	13	29.8	5.2
Cambridge_Buckner	198	123	75	21	2.3
Cleveland	31	20	11	43.5	11.1
COBRE	74	23	51	35.8	11.6
Dallas	24	12	12	42.6	20.1
ICBM	86	45	41	44.2	17.9
Leiden_2180	12	0	12	23	2.5
Leiden_2200	19	8	11	21.7	2.6
Leipzig	37	21	16	26.2	5
Milwaukee_b	46	31	15	53.6	5.8
Munchen	16	6	10	68.4	4
Newark	19	10	9	24.1	3.9
NewHaven_a	19	9	10	31	10.3
NewHaven_b	16	8	8	26.9	6.3
NewYork_b	20	12	8	29.8	9.9
Orangeburg	20	5	15	40.6	11
Oulu	103	66	37	21.5	0.6
Oxford	22	10	12	29	3.8
PaloAlto	17	15	2	32.5	8.1
Pittsburgh	17	7	10	37.9	9
Queensland	19	8	11	25.9	3.9
SaintLouis	31	17	14	25.1	2.3
TOTAL	1170	636	534	29.8	14

Preprocessing of anatomical data consisted of skullstripping and normalization to the MNI152 template (at the resolution of 1 mm isotropic). Functional data were initially motion corrected, skullstripped, and the transformation matrix to MNI152 space was computed as the product of the transformation matrix of functional data to the anatomical space and the transformation matrix obtained during anatomical preprocessing. Subsequently, all voxel time series were bandpassed (f between 0.01 and 0.08 Hz) and detrended using a linear model including a polynomial baseline and motion parameters as regressors with AFNI's 3dDeconvolve. Regional homogeneity (Zang et al., 2004) was computed on cubes of 3 × 3 × 3 = 27 voxels in native resolution, resulting maps were transformed to MNI152 space using the matrix calculated earlier, and resampled to 3 mm isotropic voxels. The choice of ReHo as the input for a classifier was based on two reasons: first, previous work has shown it to be a robust measure useful for classification of sex (Wang et al., 2012) and it can therefore be used as a meaningful baseline. Second, contrary to measures operating on a priori decompositions of the brain (e.g., connectivity between nodes based on anatomical or functional ROIs, as performed by Vergun et al., 2013), a voxelwise measure such as ReHo is entirely data driven and thus optimally suited for our purpose of examining approaches for purely automated classification.

**Figure 1:**
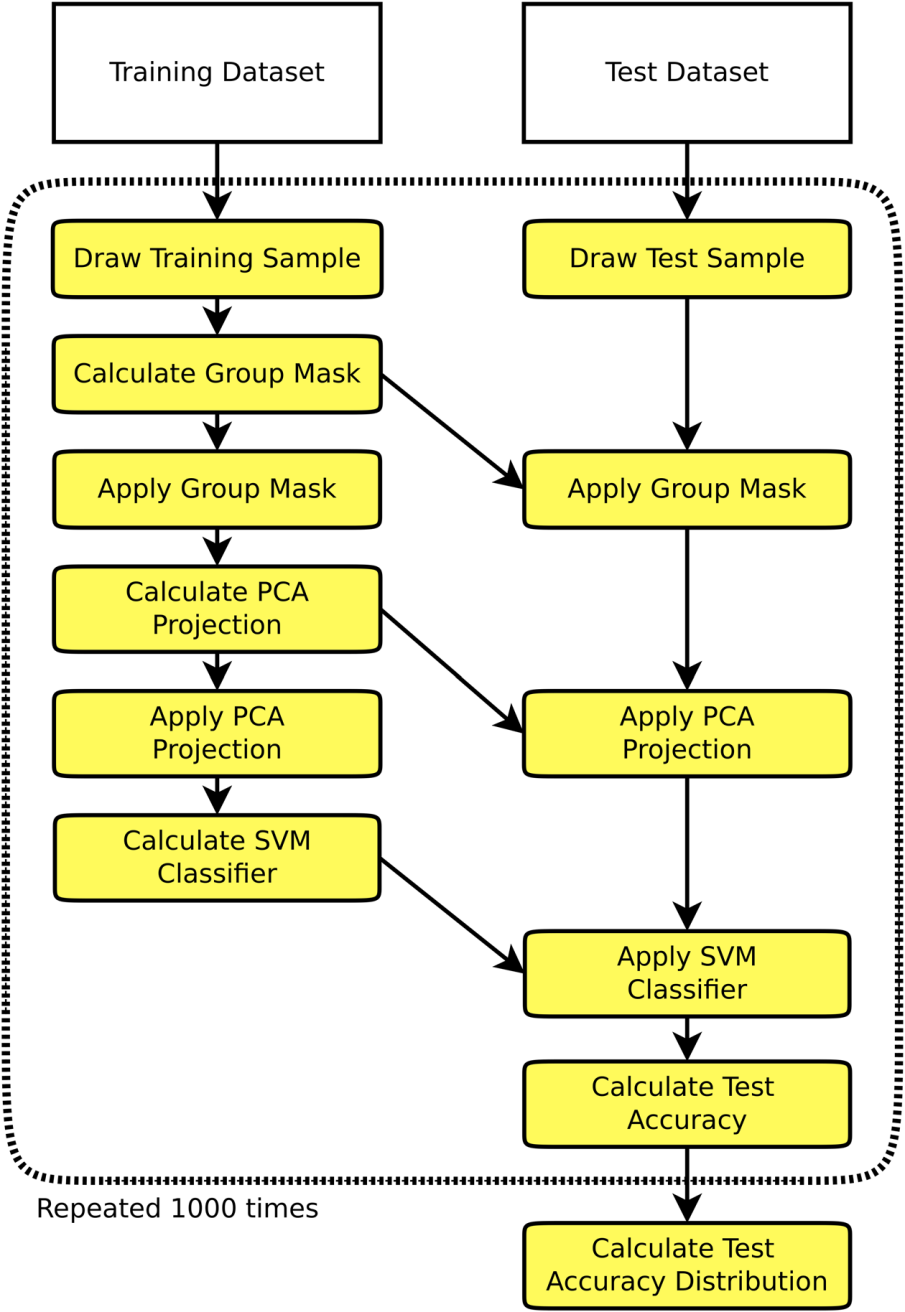
**Flowchart of one simulation run consisting of the selection of a training set and a test set independent of the training set, training a classifier on the training set, and testing the classifier on the test set**. This procedure is repeated 1000 times to derive an empiric distribution of the test accuracy for a given training sample size and a given test sample size.

On the single-subject data thus preprocessed, multiple simulation runs were performed. One simulation run (see Figure 1) consisted of the selection of a training and a test set (allowing no overlap between the two), training of a classifier on the training set, and testing of the classifier on the test set. A multitude of different training and test set configurations were chosen as described below, and each configuration was run on 1000 random samples. The training stage started with the creation of a group mask comprising all voxels included in at least 95% of all single subjects' brain masks. This kind of masking ensures that there is enough overlap across all 1170 subjects of the dataset to perform whole brain analyses. To complement the derived set of results, we also performed the above analyses for the four largest single studies in the dataset using only voxels within the individual subjects' gray matter masks instead of the whole brain masks, the results being shown in the Supplementary material. To use only information available in the training datasets, the brain mask was calculated on the training subjects only, meaning that dimensionality differed across simulation runs. The training stage proceeded with the creation of an n × p data matrix of regional homogeneity values, n being the number of subjects in the training dataset, and p being the number of voxels in the group brain mask. This data matrix was then orthogonalized using principal component analysis (PCA) without dimensionality reduction, and a support vector machine (SVM) classifier using a linear kernel was trained (Cortes and Vapnik, 1995). A linear kernel was used to reduce the potential for overfitting, this decision being supported by the encouraging results obtained by Wang et al. (2012), who also used a linear kernel SVM on ReHo data. While nonlinear kernels like radial basis functions are also a possible choice for SVM based pattern analyses, we aimed at investigating a baseline for classifier accuracy and thus opted for the more robust and established linear kernels.

The test stage consisted of the extraction of the regional homogeneity values of the relevant voxels using the mask generated during the training stage, projection of the test data onto the PCA space and application of the SVM classifier to generate predicted sex labels for the test dataset. Overall classification accuracy was then calculated as the ratio of correctly classified test subjects to the total size of the test sample. From the 1000 test sample classification accuracies, the mean and standard deviation was calculated.

The first group of simulation configurations was performed for the four largest datasets included in the FCon database (i.e., Beijing, Cambridge, ICBM, and Oulu) using in each case a single study for both the training and the test datasets. In these runs test and training sample sizes were equal, starting at 20 and using sample sizes up to half the number of subjects available when using balanced samples, increasing sample sizes in steps of 10. For example, the Beijing dataset included 118 females and 74 males, and training sample sizes of 20, 30, 40, 50, 60, and 70 were used (each sample comprising equal numbers of males and females), still allowing for equally sized test datasets (and resulting in a total of 70 males and 70 females used for one simulation—35 of each in both the training and test samples). For each sample size, 1000 random samples were drawn and the mean as well as the standard deviation of the test set classification accuracies were computed.

In a second group of simulation configurations, generalizability of a classifier trained on one dataset to a wider population was examined by sampling the test population from all subjects except those of the study used for the training dataset. Again, training datasets were Beijing, Cambridge, ICBM, and Oulu, and training sample sizes ranged from 20 to the maximum possible for each dataset by increments of 10, while still maintaining balanced samples. This led to larger sample sizes than in the first group, because the test population was not drawn from the same study. In the example of the Beijing dataset used above, this means that the maximum training sample size was 140, including 70 males and 70 females.

The third group of simulations was aimed at quantifying the generalizability of a classifier trained on one study dataset to a test sample from another single-study dataset. All pairs of test and training datasets using the four largest FCon studies as above were examined, leading to 12 pairs of training and test datasets. Training sample sizes employed were the same as in the second group of simulations, test sample sizes were always the maximum balanced sample as used above for the test study sample, i.e., 140 for Beijing and Cambridge, 80 for ICBM, and 70 for Oulu.

The fourth group of simulations investigated the performance of classifiers trained on a multi-study population on a test dataset from a single study. For the latter, the same four studies as above were used, and the training dataset in each analysis was composed of all subjects from all other (i.e., 26) studies. Training sample sizes used were all sizes ranging from 20 to 200 in increments of 10, and test sample sizes chosen represent the maximum balanced sample sizes for each study, as used in the second and third groups of simulations.

The fifth group of analyses used pooled multi-center samples for both the training and test samples. Training sample sizes varied from 20 to 200 as in the previous group and test sample sizes were 200 subjects. After performing this analysis on all subjects, a similar analysis was repeated on a sample excluding the two largest FCon studies (i.e., Beijing and Cambridge, which included 390 of the total of 1170 subjects), to eliminate the possibility of the analysis being too strongly influenced by these two large studies. Unless otherwise highlighted, all computations were performed in R version 3.0.2 (R Development Core Team, 2013).

## 3. Results

In general terms, classification accuracies obtained were mostly above 0.5 and reached levels up to about 0.8, with higher training sample sizes leading to higher accuracies.

**Figure 2:**
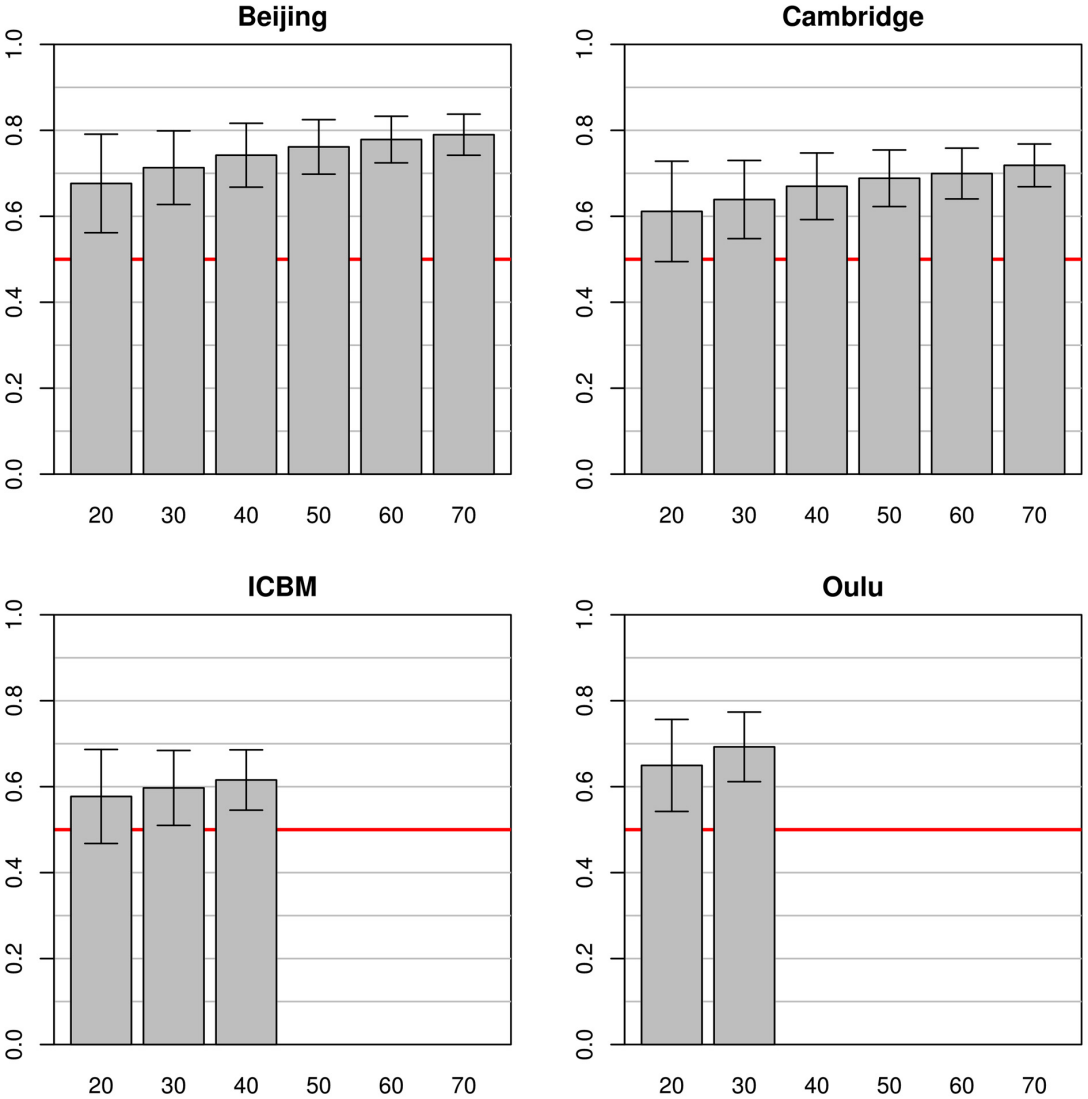
**Simulations using equally sized test and training datasets from the same study**. Each bar represents the mean test accuracy for a particular sample size (which was used for both the training and test samples) and the whiskers represent the respective standard deviations. The red line indicates 0.5 accuracy, as by chance.

In the simulations using data from a single study as a source for both training and test datasets (see Figure 2), marked differences between the simulation results from the four source datasets were found. The best classification accuracy on the test datasets was obtained with the Beijing dataset, leading to mean classification accuracies from 0.68 to 0.79 with training sample sizes of 20 and 70, respectively. The second best accuracy was achieved on the Oulu dataset where only sample sizes of 20 and 30 could be used for simulations, with a maximum classification accuracy of 0.69. On the Cambridge dataset classification accuracies varied from 0.61 to 0.72 for sample sizes ranging from 20 to 70. Finally, the ICBM dataset showed lowest test dataset classification accuracies of 0.58 to 0.62, still clearly above 0.50 (i.e., chance accuracy). In all four cases, the standard deviations of the test set accuracies were lower with higher sample sizes. For example, in the Beijing study standard deviation for sample size 20 was 0.11 but could be reduced to 0.05 by increasing the sample size to 70.

**Figure 3:**
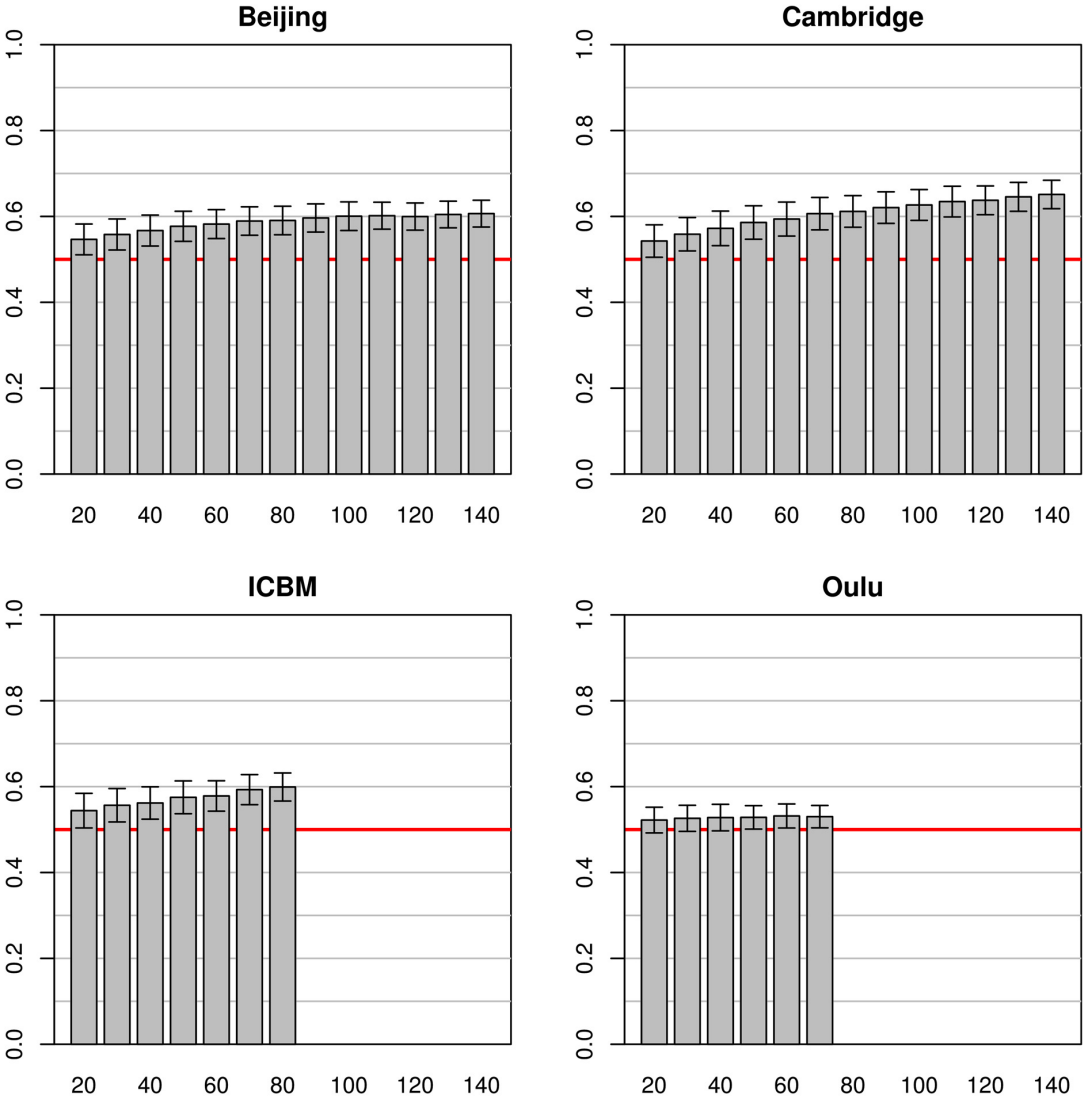
**Simulations using subjects from one study for the training datasets (with varying sample sizes) and 200 subjects from all other studies for the test datasets**. The bars and whiskers represent means and standard deviations of test accuracies as in Figure 2. Note that while Beijing and Oulu exhibit high accuracies in Figure 2 they tend to show lower accuracies than the other two studies in this analysis.

When using the whole pooled dataset as a source for the test sets (instead of using the same study as a source for both training and test sets) the results observed were quite different (see Figure 3). The best test accuracy could be obtained when using the Cambridge dataset as the source for the training data with test set accuracies from 0.54 (on a training sample size of 20) to 0.65 (on a training sample size of 140). Beijing and ICBM datasets provided comparable results, both yielding an accuracy of 0.55 and 0.54, respectively, with training sample sizes of 20 and accuracies of 0.59 and 0.60, respectively, for training sample sizes of 80 subjects. When using the largest sample size of 140, the test accuracy of the Beijing classifier was 0.61, slightly below the accuracy obtained using the Cambridge dataset. The simulations on the Oulu dataset achieved the lowest test accuracy of 0.52 and 0.53 for training sample sizes of 20 and 70, respectively. In contrast to the simulations using the same studies for both training and test datasets, standard deviations of mean accuracies were lower and relatively constant across all training sample sizes, and even across studies (range 0.026 to 0.040).

**Figure 4:**
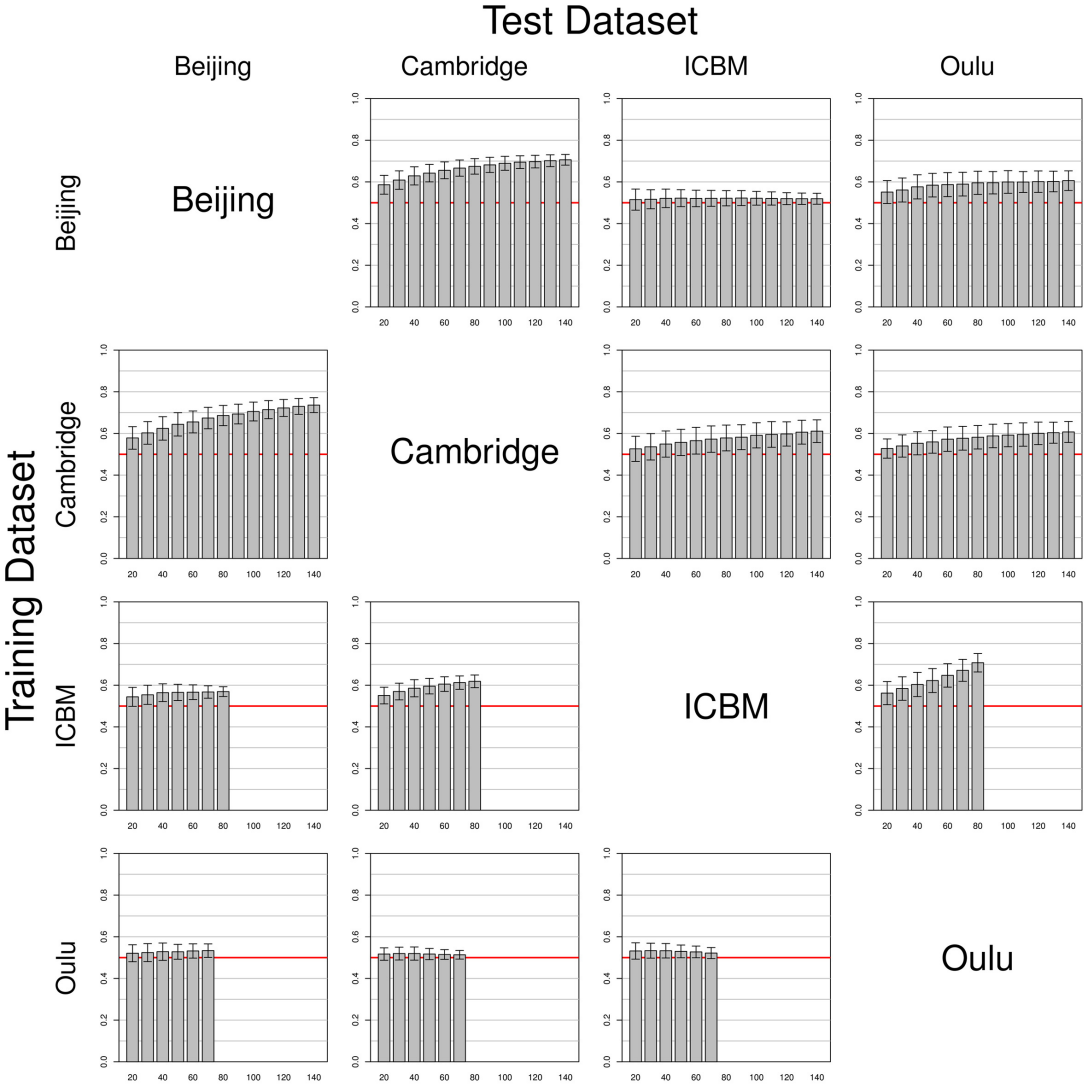
**Simulations using test and training datasets from two different studies among the four largest FCon datasets, with each row having the same study as a source for training datasets (varying training dataset size) and each column having the same study as a source for the test datasets (fixed test sample size, see section 2)**. Bars and whiskers represent means and standard deviations of test accuracies. Note the asymmetric behaviors of some pairings (e.g., Beijing/ICBM vs. ICBM/Beijing, and how the Oulu dataset yielded higher accuracies when used as a test dataset as opposed to being used as a training dataset).

Simulations using data from two different single studies as training and test datasets (one for each set) yielded mixed results (see Figure 4). While some pairs of studies seem to work well together (e.g., Beijing and Cambridge), others yielded results that were undistinguishable from pure chance (e.g., Beijing as training dataset and ICBM as test dataset). It should be noted that results were not symmetric regarding test and training datasets. For example, in contrast to the result just described, when using ICBM as training dataset and Beijing as test dataset, classifiers with a classification accuracy consistently >0.5 could be obtained. Another interesting example is the asymmetry between Oulu yielding only low classifier accuracy when used as a training dataset, but yielding much better accuracies when used as a test dataset. Overall, classifier accuracy was consistently above chance level in all combinations except the Beijing/ICBM pairing mentioned above, with accuracies varying between slightly above 0.5 (Oulu as training dataset) and about 0.75 (Cambridge as training dataset and Beijing as test dataset).

Note that increases in sample size of the training dataset most often led to an increase in classifier accuracy, but the extent of this increase varied across studies. For example, a continuous increase of classifier accuracy with increasing sample size was seen with the pairing Cambridge/Beijing. In other cases no consistent trend in accuracy over different sample sizes could be observed, notably when using the Oulu dataset as training dataset. A third case observed was that accuracy increased with sample size almost asymptotically until increases in training dataset size led to no further increases in accuracy on the test dataset (e.g., Beijing/Oulu). Concerning the variance, with most pairings an increase in training sample size led to a decrease in variance of the accuracy on the test dataset, though counterexamples were also found (e.g., the combination Cambridge/ICBM) where mean accuracy increased with increasing training sample size but variance remained approximately constant. Results from the analyses using the gray matter masks instead of the whole brain masks are largely similar, see Figures S1–S3 in the Supplementary material for the analyses corresponding to Figures 2–4.

**Figure 5:**
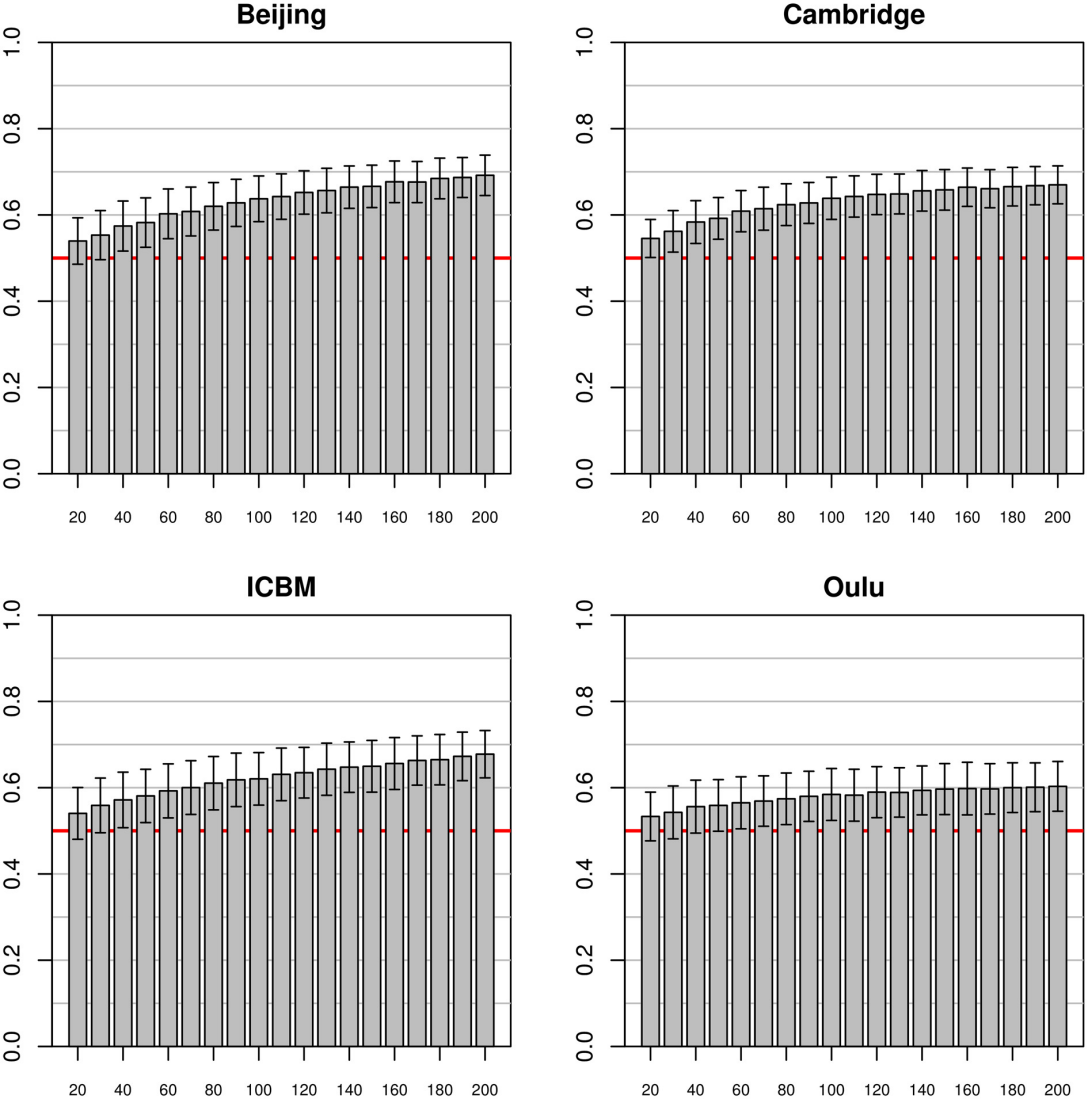
**Simulations using training datasets from the pooled sample and using subjects from a single study as test dataset**. Bars and whiskers represent means and standard deviations of the test accuracies. Mean test accuracies for the Beijing, Cambridge and ICBM datasets appeared to asymptotically approach about 0.68, while accuracies using the Oulu dataset seemed to level out at a lower levels of about 0.6.

In simulations using the classifier trained on the pooled dataset excluding one of the four largest FCon studies which was subsequently used as test sample (see Figure 5), mean accuracies seemed to asymptotically approach about 0.68 for the Beijing, Cambridge and ICBM test samples, while the standard deviation remained largely constant across sample sizes. When using the Oulu dataset as test dataset, mean accuracies where consistently above 0.5 for all sample sizes but markedly lower than for the other three studies, with a relatively large amount of individual simulation runs leading to a test accuracy below 0.5.

**Figure 6:**
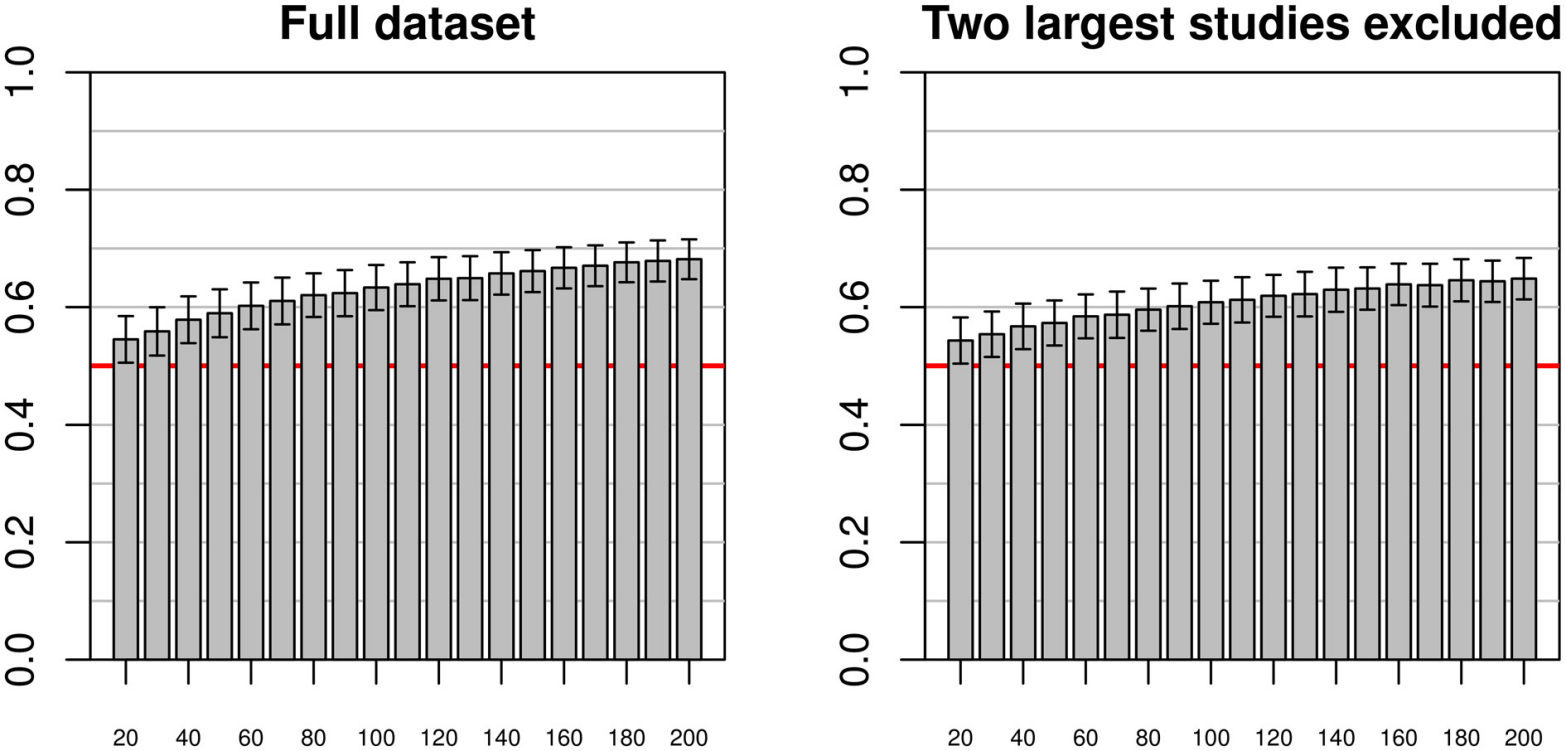
**Simulations using test and training datasets from the pooled sample of all studies (left) and a pooled sample of all but the two largest studies (right) using varying training sample sizes between 20 and 200, and a constant test sample size of 200**. As with the previous figures, bars and whiskers represent means and standard deviations of test accuracies. In both cases mean accuracies approached 0.6 with increasing training sample size, with mean accuracies on the reduced dataset slightly below the mean accuracies of the complete dataset.

When using the entire pooled dataset as training and test dataset (see Figure 6), mean accuracies of about 0.68 could be achieved, with relatively low standard deviations of about 0.034. There appeared to be asymptotic behavior of the mean accuracies with increasing training sample size, while the standard deviation remained relatively constant across sample sizes. In the second analysis, excluding the two largest individual studies (Beijing and Cambridge), the results were similar but the convergence toward the maximum seemed slower (indeed, the maximum mean accuracy achieved was 0.65).

**Figure 7:**
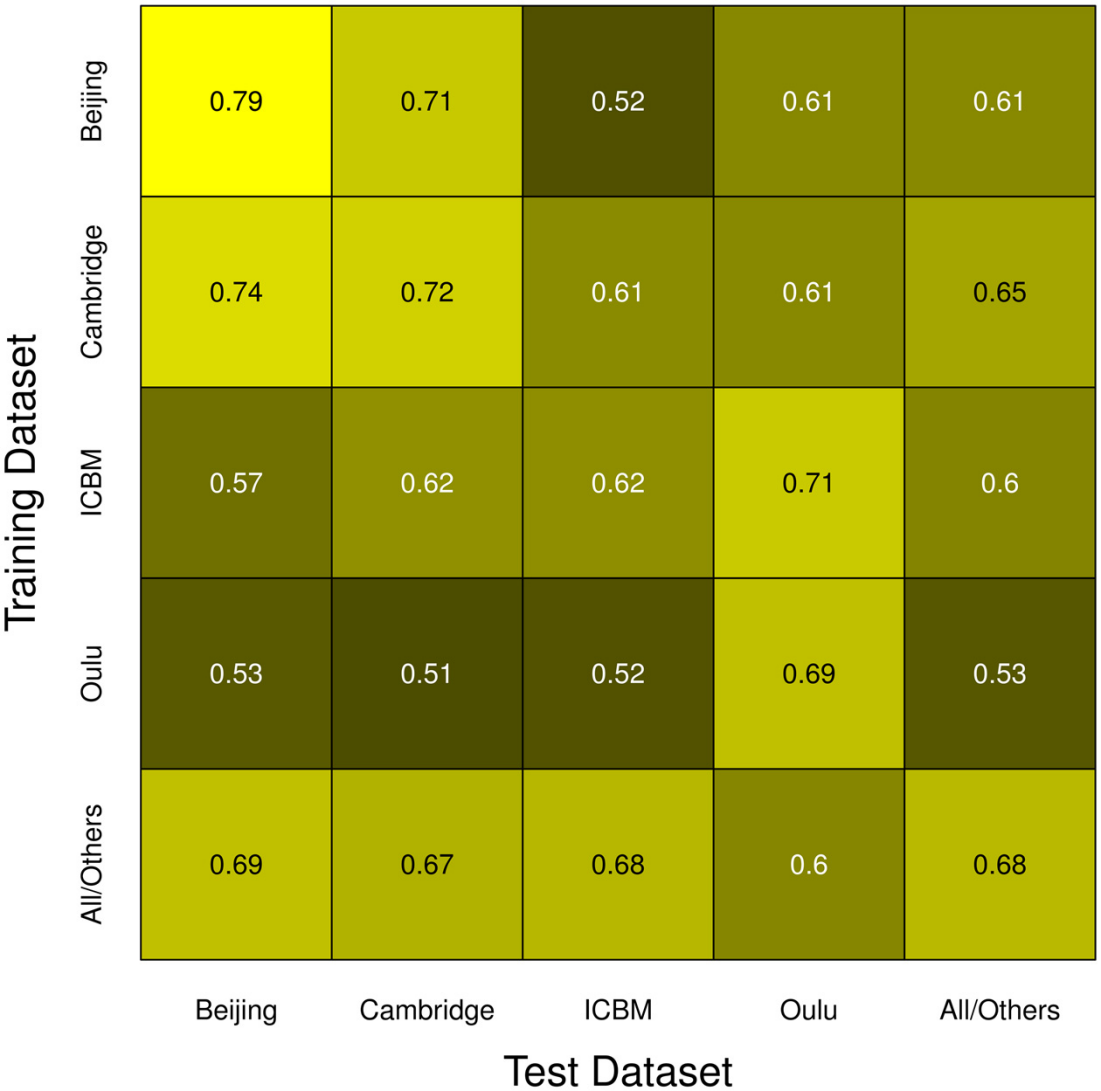
**Summary of all simulation runs**. The color of each cell represents the mean accuracy of the respective test/training dataset pair's simulation run with the largest of the sample sizes used in that comparison, with brighter colors corresponding to higher test accuracies. Note the highest accuracies being located in the main diagonal, as well as the asymmetry in the off-diagonal cells.

Overall, the highest test accuracies could be achieved when using training and test samples from the same study as well as in some particular combinations of studies (e.g., Beijing as training and Cambridge as test dataset and vice versa). Figure 7 summarizes the mean accuracy achieved with the maximum sample size for all pairs of studies, including a category “All/Others” meaning pooled samples as described in the Methods section, with brighter colors indicating higher accuracies. Note that sample sizes in the main diagonal were half the sample sizes in off-diagonal fields except for the “All/Others” category. In this depiction, it can easily be seen that the Beijing/Cambridge combinations yielded highest accuracies with other high accuracies achieved in the Oulu/Oulu, ICBM/Oulu, and Cambridge/Others pairs. The lowest accuracies were obtained when using Oulu for training and another dataset for testing, as well as in the particular combination Beijing/ICBM already highlighted above.

**Figure 8:**
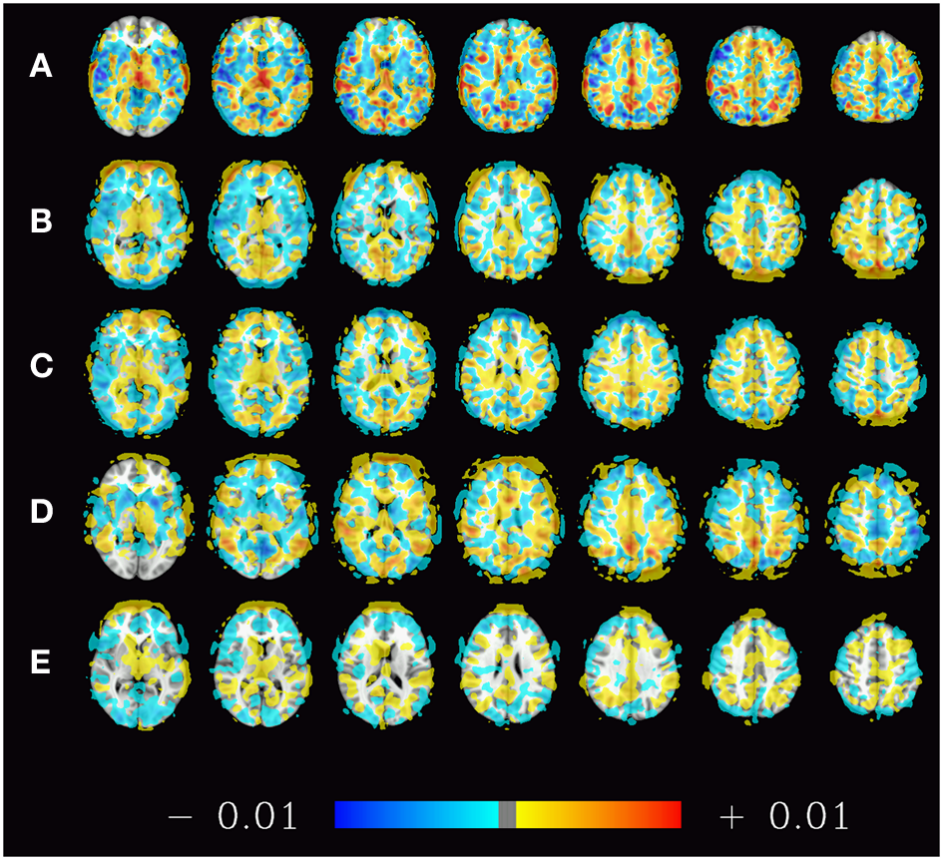
**Spatial distribution (mean weights) of voxels contributing to the 1000 classifiers trained on the training samples of the pooled dataset (A), the datasets of Beijing (B), Cambridge (C), ICBM (D), and Oulu (E) with sample sizes of 200, 70, 70, 40, and 30 subjects, respectively**. The maps presented thus correspond to the rightmost bars in the subfigures of Figure 2 as well as the left subfigure of Figure 6. Positive weights indicate higher ReHo values in males, negative weights indicate higher ReHo values in females.

For illustration purposes the spatial distribution of voxels contributing to the classifier can be projected back into the original space. This has been performed for the estimators trained on the subsets of 70 subjects from Beijing dataset that produced the results in Figure 2 (top left). The mean of all 1000 such maps computed during this simulation is shown in Figure 8 to give an idea about how the classifiers employed here weigh certain parts of the brain.

## 4. Discussion

This study evaluated the generalizability of machine learning estimators in fMRI studies, based on four criteria. First, we evaluated how well a classifier trained on a single-study dataset could be applied to a dataset from a different study. Second, generalizability of such a classifier to a wider population was investigated using a pooled multi-center dataset. The third criterion for the generalizability of machine-learning estimators was the performance of a classifier trained on a pooled multi-center dataset to a particular single-study dataset. Finally, the last criterion was how accurately a multi-center classifier could classify data from an equally wide multi-center population. The key result from these analyses is that generalizability cannot adequately be assessed using data originating from a single site alone, and that a multi-center dataset is needed to quantify the broader generalizability of a machine learning classifier.

In general, mean test accuracies increased with increasing training sample size, though there were some exceptions to this. The variance of the test accuracies decreased with increasing sample size in some groups of simulations (e.g., single-study training sets with single-study test sets), but remained about constant across all sample sizes in other groups of simulations, e.g., most simulations involving pooled samples (see Figures 3, 5). In single-study analyses, when using a test sample from the same dataset as the training set mean accuracies were higher than when using combinations of training and test datasets from two distinct studies. This reflects the common situation that a small group of subjects, homogeneously selected and scanned, may allow the detection of small but significant differences most easily, of course with the caveat of low generalizability. When using two distinct studies for training and test datasets, there is a clear asymmetry in results in that one of the pairs might yield higher test accuracies than the other (see Beijing/ICBM vs. ICBM/Beijing in Figures 4, 7). The analyses using classifiers trained on the pooled sample (see Figures 5, 6) tended to converge to about 0.65 with increasing training sample size.

It is noteworthy that the four studies analyzed separately yielded quite different patterns of results. Studies with high within-study test accuracy (that is, the accuracy achieved with test and training dataset drawn from the same study) tended to have stronger decreases in accuracy when generalizing to a test dataset drawn from different studies (both when relating to single-study and pooled test datasets). Some studies yielded higher accuracies when used as training dataset (see ICBM in Figure 4) while others had higher accuracies when used as test dataset (see Oulu in Figure 4).

**Figure 9:**
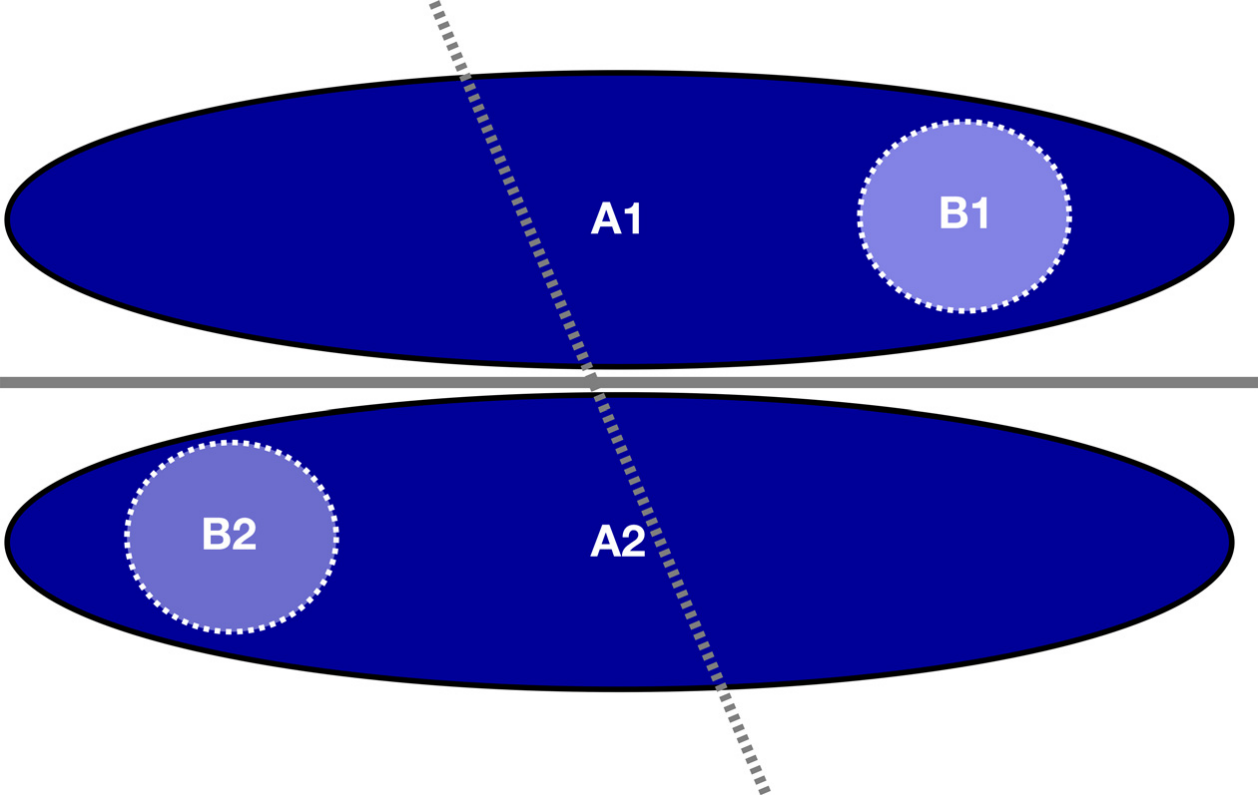
**Example of how a classifier on a subset of the total sample (light blue circles, dotted line) fails to adequately classify the total sample (dark blue ellipsoids, solid line) though the latter can be classified by itself, even yielding perfect classification accuracy on the smaller subset**.

While these findings might seem counterintuitive at first, they can be explained in the following way. A homogeneous single-study dataset can be seen as a subset of a wider population covering only a limited part of the input data space of the total population. If a classifier exists that can perfectly separate the groups in the total population, than this classifier can also perfectly separate the groups in the subset. In the opposite case a classifier which can separate the groups in a subset might not be able to separate the groups in the total population (see Figure 9).

It can be speculated that one possible reason behind high homogeneity in the classifier results emerging from the Beijing and Oulu datasets as compared to those from the Cambridge and ICBM datasets might be a more homogenous study population (in addition to a range of potential technical reasons). Indeed, the Oulu and Beijing datasets have the two smallest standard deviations of the age of the participants, while the ICBM dataset had the third largest age standard deviation of all 35 original studies included in the dataset used (see Table 1). On a more conjectural note, both Cambridge and ICBM have been acquired in the United States, likely based on a genetically more versatile pool, as have been about 700 out of 1170 subjects in the entire sample, which might also explain why these two study datasets yield better generalization results on the total sample. Similar effects might explain the asymmetry in Figure 4.

When aiming at generalizability one might ask what the optimal classification accuracy that is achievable with a particular classifier on particular data would be. The best estimator for this optimal classification accuracy can be found in the simulations including the total sample. Both when testing single-study classifiers or classifiers estimated at the pooled dataset on a test dataset from the pooled sample results seemed to converge to a test accuracy of about 0.65 in a consistent manner. This accuracy was found irrespective of whether the two largest studies were excluded from the sample or not (see Figure 6), corroborating the robustness of this finding which thus seems not to be driven only or mainly by the two largest studies. One might consider this value as the maximum achievable with a linear SVM on regional homogeneity data preprocessed as presented in this study.

When compared to results reported from single studies a classification accuracy of 0.65 appears to be somewhat underwhelming. Our simulations might thus point to the disappointing conclusion that even a classifier that worked well for one particular dataset (e.g., the dataset on which it was trained and with which it was published) might fail to classify with good accuracy on a different dataset. A relatively low classification accuracy of a resting-state machine learning classifier is not particularly surprising though: a 2012 competition aiming at finding the best possible model for classifying attention deficit hyperactivity disorder (ADHD) patients revealed that no fMRI based classifier submitted by any of the participating groups could outperform a classifier based on clinical variables alone (Brown et al., 2012), which achieved a classification accuracy of 0.63 (chance accuracy being 0.55 in this case).

To address this type of concern it seems most helpful to take a step back and ask what the goal of the classifier originally was. In some cases generalizability to other clinical populations is not a required feature for the classifier to be applicable in practice. For example, a clinical application might be to classify between positive and negative outcomes within a particular at-risk population and, therefore, it is not necessary for the classifier to yield plausible results in a broader population outside these at-risk groups. It might also be required in practice to modify and train the classifier for a particular population (e.g., the particular population at a certain hospital site) and a single classifier does not need to be applicable to all populations when this specific classifier training can be performed. Even if a general classifier is able to classify correctly with an acceptable reliability it might be helpful to further optimize this classifier on a particular hospital site to maximize its accuracy within the specific boundaries in which it will be applied in practice.

On the other hand, a generalizable classifier might be useful for some neuroscientific research questions even if its accuracy is not very high. In this case it might not be of use for diagnostic purposes but the classifier itself could give investigators clues on physiological or pathological patterns underlying the groups to be classified.

For practical reasons most machine learning fMRI studies include a sample acquired on one site only and thus provide no means for accurately estimating the across-study generalizability of their classifier. In some cases an estimation can be achieved by testing the classifier on a publicly available multicenter dataset, though this might not always be sensible or even possible. Among other reasons, for a machine learning study investigating the classification of a particular clinical variable no public dataset including this clinical variable might be available. In addition, the testing of generalizability of machine learning classifiers on public datasets—which are the same for all studies using this method of evaluation—might introduce another form of bias, namely overfitting to the test dataset. Thus, an evaluation of generalizability using always the same set of public datasets might not be advisable in the long term.

There might be some choices in study design to consider that help to improve generalizability for researchers. It seems that, in general, larger sample size leads to increased test accuracy, both within the same dataset and when applying the classifier to a different dataset. Furthermore, choosing a sample that is biologically not too homogeneous might give results being more representative of a wider population, if generalizability is one of the aims of the study. If on the other hand a maximum classifier accuracy on a particular sample is the goal of the study, a homogeneous study sample might yield better results; the exact composition of the sample should then be clearly described to allow the reader to assess the range of applicability of the classifier. In any case, technical variability in image data collection should be as low as possible, controlled by standardized quality control procedures.

One of the main results of the present study is quantification of the relationship between the accuracy of a classifier on the dataset which it was trained on and its generalizability to different datasets. Our results show that even when a classifier achieves up to 80% test accuracy on its own study population (even if it does not overfit to the training data), the test accuracy on a more general population might realistically drop to 65% or less (in this specific case with balanced samples). While an increase in sample size of the training dataset generally leads to higher test accuracies, these accuracies are more dependent on other factors (the similarity between the samples might be one of them when investigating across-study generalizability). Thus, even a very large sample size cannot guarantee that a particular machine learning classifier is suitable for new data (be it a different study or clinical populations). A practical approach for scientists planning to use a published classifier should thus include a pilot study to investigate how well this classifier can be generalized to one's own dataset (study population, technical setup etc.).

To conclude, our results indicate that researchers performing machine-learning studies need to consider generalizability of their classifier separately from its accuracy on their own dataset. This generalizability can only be assessed using different data, ideally from one or more different study sites. Overly optimistic classifier accuracies reported should be taken with a grain of salt, since practical generalizability of resting-state fMRI machine-learning classifiers is still relatively low (Brown et al., 2012). Instead of indulging in the use of more complex classifiers on datasets that are inherently difficult to separate, it is advisable to first examine the separability of the dataset itself with relatively robust methods, yielding a conservative estimate of what can plausibly be achieved.

### Conflict of interest statement

The authors declare that the research was conducted in the absence of any commercial or financial relationships that could be construed as a potential conflict of interest.
