# Supplementary material for: On the generalizability of resting-state fMRI machine learning classifiers
Source: Front Hum Neurosci. 2014 Jul 29;8:502. doi: 10.3389/fnhum.2014.00502 (PMC4114329; doi:10.3389/fnhum.2014.00502)
Supplement: Supplementary file 1 [file Presentation1.PDF]

On the Generalizability of Resting-State fMRI Machine Learning Classifiers

Supplement

Huf W[abc*], Kalcher K[abc*], Boubela RN[abc], Rath G[bd], Vecsei A[e], Filzmoser P[c], Moser E[abf]

[a] *Center for Medical Physics and Biomedical Engineering, Medical University of Vienna, Währinger Gürtel 18-20, 1090 Vienna, Austria*

[b] *MR Centre of Excellence, Medical University of Vienna, Lazarettgasse 14, 1090 Vienna, Austria*

[c] *Department of Statistics and Probability Theory, Vienna University of Technology, Wiedner Hauptstraße 8-10, 1040 Vienna, Austria*

[d] *Department of Radiodiagnostics and Nuclear Medicine, Medical University of Vienna, Währinger Gürtel 18-20, 1090 Vienna, Austria*

[e] *St. Anna Children's Hospital, Department of Pediatrics and Adolescent Medicine, Medical University of Vienna, Kinderspitalgasse 6, 1090 Vienna, Austria*

[f] *Department of Psychiatry, University of Pennsylvania Medical Center, 10, Gates Bldg., Philadelphia, PA 19143, USA*

[*] *Wolfgang Huf and Klaudius Kalcher contributed equally to this work.*

July 7, 2014

Supplement

To assess the influence of masking on the machine learning results of the simulations that underlie figures 2, 3, and 4 we re-ran those simulations with an additional preprocessing step: after the preprocessing described above and before the drawing of training and test samples, we introduced another step consisting of masking for each subject the ReHo dataset with the respective gray matter mask as obtained by automatic segmentation of the anatomical image by FSL FAST. Results are presented in figures S1, S2 and S3. Mostly, results are similar to the results seen in the original simulations, with a few minor deviations. In figure S1, the classification accuracies for the Beijing and Oulu datasets are lower than the corresponding accuracies in figure 2, while the accuracies on the Cambridge and ICBM datasets remain approximately constant. Figure S2 remains essentially the same as figure 3, and in figure S3 the results for the train/test pairs Beijing/ICBM and Beijing/Oulu show an increase in classification accuracy, while the results on the pairs Cambridge/Oulu and ICBM/Oulu exhibit a decrease in accuracy. Thus, the most notable difference between the two sets of results is the increase in generalizability of the classifiers trained on the Beijing dataset at the cost of decreased test accuracy within its own population. Overall, at least for the four big studies in the 1000 Functional Connectomes project, the results with gray matter masking are remarkably similar to the results without gray matter masking, suggesting that the influence of non-gray matter voxels on the overall classification accuracy is rather limited.

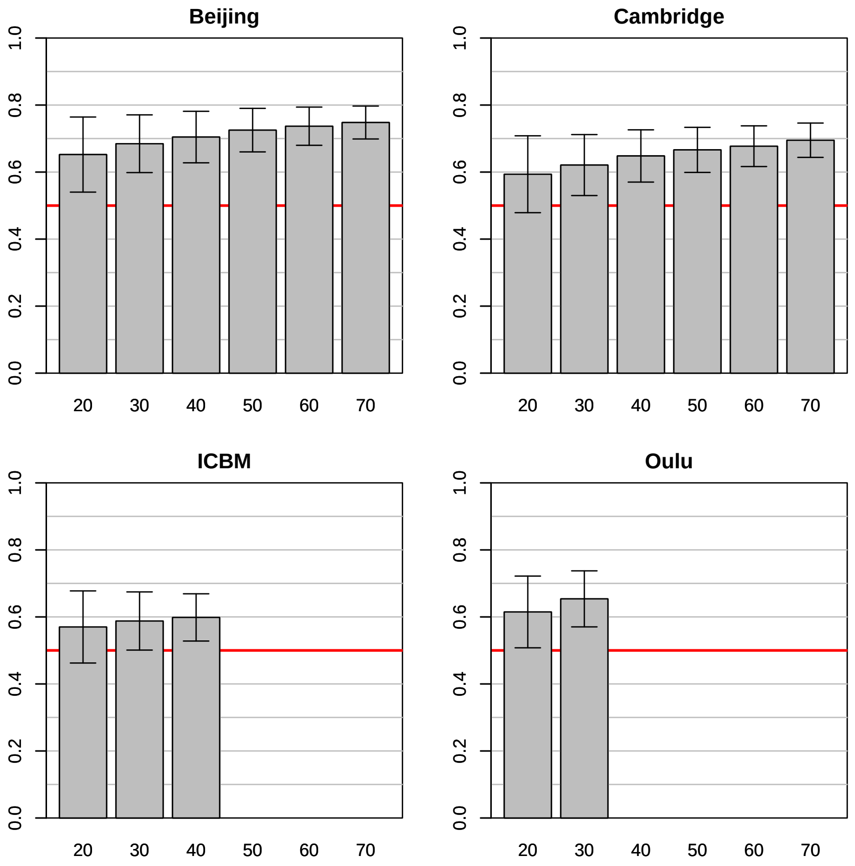

Figure S1: Simulations using equally sized test and training datasets from the same study similar to figure 2, but with the application of a gray matter mask.

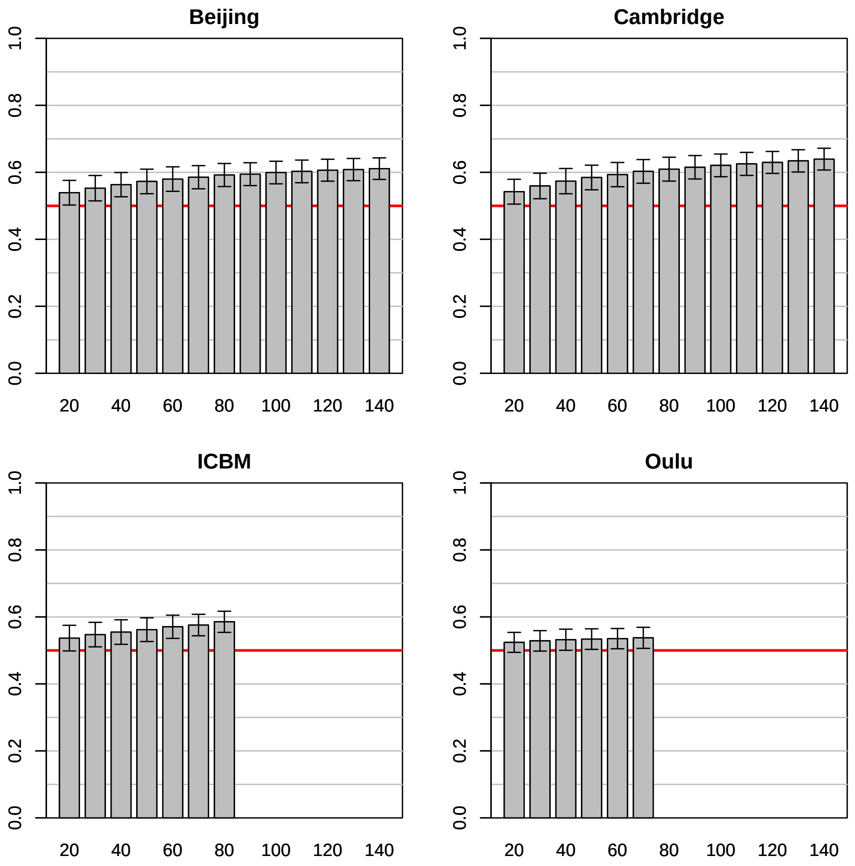

Figure S2: Simulations using subjects from one study for the training datasets (with varying sample sizes) and 200 subjects from all other studies for the test datasets, similar to figure 3, but with the application of a gray matter mask.

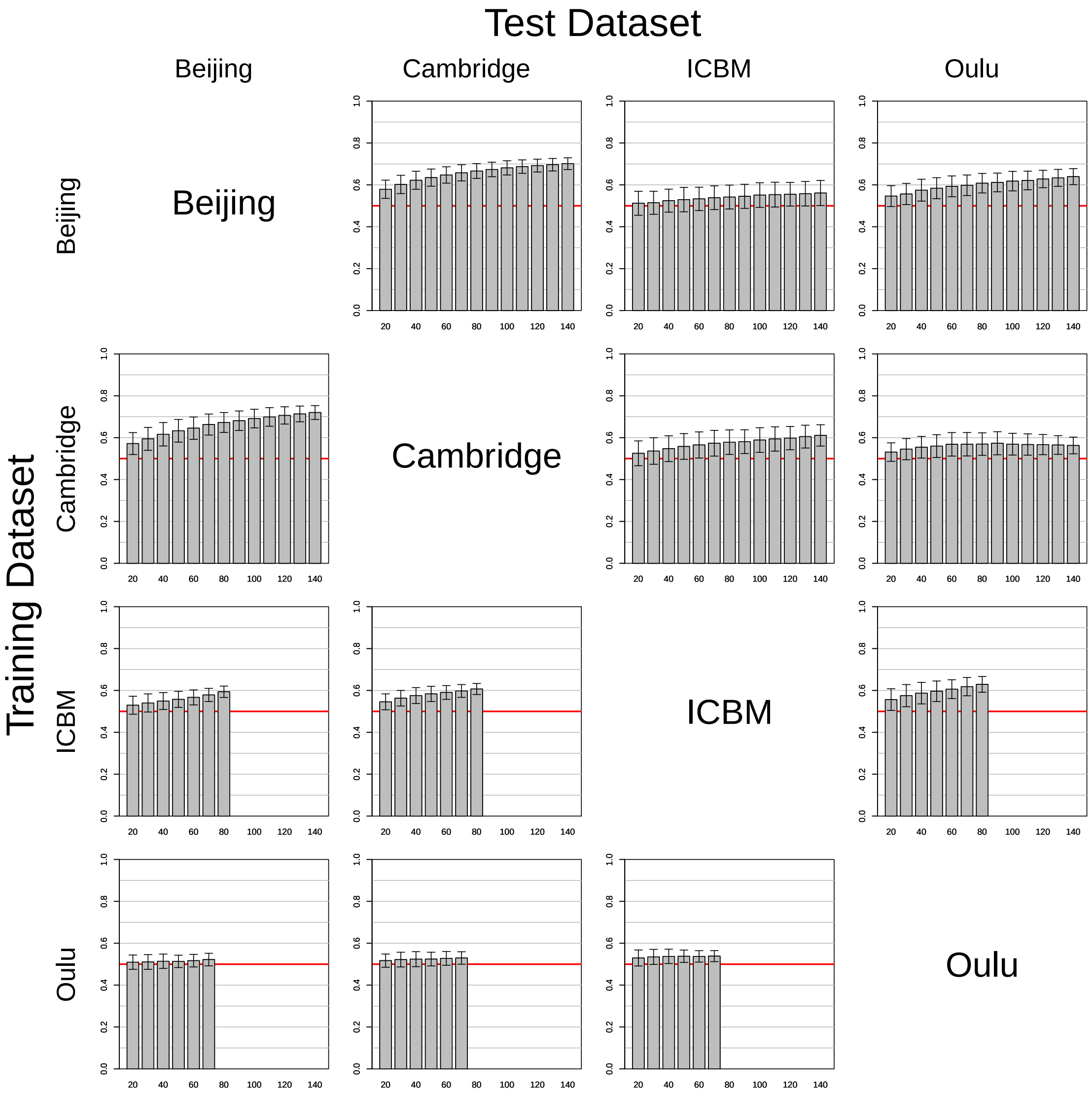

Figure S3: Simulations using test and training datasets from two different studies among the four largest FCon datasets, with each row having the same study as a source for training datasets (varying training dataset size) and each column having the same study as a source for the test datasets (fixed test sample size, see section 2), similar to figure 4, but with the application of a gray matter mask.
